# Identification and Characterization of Putative Translocated Effector Proteins of the *Edwardsiella ictaluri* Type III Secretion System

**DOI:** 10.1128/mSphere.00039-16

**Published:** 2016-05-11

**Authors:** Lidiya P. Dubytska, Matthew L. Rogge, Ronald L. Thune

**Affiliations:** a Department of Pathobiological Sciences, School of Veterinary Medicine, Louisiana State University, Baton Rouge, Louisiana, USA; b School of Animal Science, Louisiana State University Agricultural Center, Louisiana State University, Baton Rouge, Louisiana, USA; Swiss Federal Institute of Technology, Lausanne, Switzerland

**Keywords:** *Edwardsiella ictaluri*, type III secretion, translocation, effector

## Abstract

*Edwardsiella ictaluri*, a major pathogen in channel catfish aquaculture, encodes a type III secretion system (T3SS) that is essential for intracellular replication and virulence. Previous work identified three putative T3SS effectors in *E. ictaluri*, and *in silico* analysis of the *E. ictaluri* genome identified six additional putative effectors, all located on the chromosome outside the T3SS pathogenicity island. To establish active translocation by the T3SS, we constructed translational fusions of each effector to the amino-terminal adenylate cyclase (AC) domain of the *Bordetella pertussis* adenylate cyclase toxin CyaA. When translocated through the membrane of the *Edwardsiella*-containing vacuole (ECV), the cyclic AMP produced by the AC domain in the presence of calmodulin in the host cell cytoplasm can be measured. Results showed that all nine effectors were translocated from *E. ictaluri* in the ECV to the cytoplasm of the host cells in the wild-type strain but not in a T3SS mutant, indicating that translocation is dependent on the T3SS machinery. This confirms that the *E. ictaluri* T3SS is similar to the *Salmonella* pathogenicity island 2 T3SS in that it translocates effectors through the membrane of the bacterial vacuole directly into the host cell cytoplasm. Additional work demonstrated that both initial acidification and subsequent neutralization of the ECV were necessary for effector translocation, except for two of them that did not require neutralization. Single-gene mutants constructed for seven of the individual effectors were all attenuated for replication in CCO cells, but only three were replication deficient in head kidney-derived macrophages (HKDM).

**IMPORTANCE** The bacterial pathogen *Edwardsiella ictaluri* causes enteric septicemia of catfish (ESC), an economically significant disease of farm-raised channel catfish. Commercial catfish production accounts for the majority of the total fin fish aquaculture in the United States, with almost 300,000 tons produced annually, and ESC is the leading cause of disease loss in the industry. We have demonstrated the survival and replication of *E. ictaluri* within channel catfish cells and identified a secretion system that is essential for *E. ictaluri* intracellular replication and virulence. We have also identified nine proteins encoded in the *E. ictaluri* genome that we believe are actively transferred from the bacterium to the cytoplasm of the host cell and act to manipulate host cell physiology to the advantage of the bacterium. The data presented here confirm that the proteins are actually transferred during an infection, which will lead to further work on approaches to preventing or controlling ESC.

## Abstract (precis)

The bacterial pathogen *Edwardsiella ictaluri* causes enteric septicemia of catfish (ESC), an economically significant disease of farm-raised channel catfish. Commercial catfish production accounts for the majority of the total fin fish aquaculture in the United States, with almost 300,000 tons produced annually, and ESC is the leading cause of disease loss in the industry. We have demonstrated the survival and replication of *E. ictaluri* within channel catfish cells and identified a secretion system that is essential for *E. ictaluri* intracellular replication and virulence. We have also identified nine proteins encoded in the *E. ictaluri* genome that we believe are actively transferred from the bacterium to the cytoplasm of the host cell and act to manipulate host cell physiology to the advantage of the bacterium. The data presented here confirm that the proteins are actually transferred during an infection, which will lead to further work on approaches to preventing or controlling ESC.

## INTRODUCTION

The Gram-negative bacterial pathogen *Edwardsiella ictaluri* causes enteric septicemia of catfish (ESC), an economically significant disease of farm-raised channel catfish, *Ictalurus punctatus*. Commercial catfish production accounts for 85 to 90% of the total fin fish aquaculture in the United States, with about 300 million lbs. produced in 2014 (1). *Edwardsiella ictaluri* is the leading cause of disease loss in the catfish industry, accounting for an estimated 20% loss in 2009 (2). The survival and replication of *E. ictaluri* in channel catfish head kidney-derived macrophages (HKDM) (3) and a channel catfish ovary (CCO) cell line (4) were reported (5), and a type III secretion system (T3SS) that is essential for *E. ictaluri* virulence and intracellular replication was identified (5). Type III secretion systems are complex protein machines that form a needle-like structure that is able to translocate effector proteins across both the Gram-negative cell wall and the host cell membrane, directly from the bacterial cytoplasm to the cytosol of the host cell (6–10). Although the structural proteins of T3SSs are quite conserved in bacterial pathogens, the arsenal of translocated effector proteins delivered to the host is unique to each system. Thus, the effect that a T3SS has on the host varies depending on the pathogen in question, and pathogenesis is defined by the particular set of effectors produced by that pathogen. Reported T3SS functions range from intracellular uptake, surface colonization of the cell without uptake, adherence to macrophages, and inhibition of phagocytosis, cytotoxicity, vesicular trafficking, programed cell death, and up- or downregulation of inflammatory cytokines and gene expression (8, 11, 12).

Most T3SSs translocate effectors from outside the host cell across the cell membrane into the cytoplasm. Like the *Salmonella* pathogenicity island 2 (PAI 2) T3SS, however, the *E. ictaluri* T3SS translocates effectors to the host cell cytosol through the vacuolar membrane. Previous work to evaluate the development of the *E. ictaluri*-containing vacuole (ECV) showed that acidification of the ECV by vacuolar H^+^ ATPases was required for *E. ictaluri* to replicate in HKDM (13, 14). Acidification also resulted in activation of the *E. ictaluri* urease enzyme, which utilized urea produced by the HKDM-encoded arginase enzyme to produce ammonia, which resulted in subsequent neutralization of the ECV (14, 15). Both acidification and subsequent neutralization of the ECV are required for *E. ictaluri* to replicate in HKDM (13, 15).

Three putative effectors are reported for *E. ictaluri*, one in the T3SS PAI (5) and one on each of the *E. ictaluri* plasmids pEI1 (16) and pEI2 (5). Given the importance of T3SS effectors to virulence in other bacterial pathogens and the requirement for an intact T3SS for *E. ictaluri* virulence, an *in silico* study was conducted to identify additional effectors and to identify conserved domains and motifs that provided insight into possible function. Then, to evaluate active translocation of the putative *E. ictaluri* effectors to the cytosol of HKDM and CCO cells and to examine the possible role of ECV pH changes on translocation, we constructed translational fusions to the amino-terminal adenylate cyclase (AC) domain of the *Bordetella pertussis* adenylate cyclase toxin CyaA. The AC domain of the CyaA toxin was used as a reporter to demonstrate type III translocation of effector proteins in a number of Gram-negative pathogens, and this process is the method of choice for studying the translocation of T3SS effectors (17–20). The assay is based on the measurement of cyclic AMP (cAMP) produced by the interaction of the translocated AC domain and calmodulin, which is present in the HKDM cytoplasm but not in the ECV. The primary objectives of this study were to identify additional T3SS effectors of *E. ictaluri*, demonstrate active translocation of all known *E. ictaluri* effectors by the T3SS apparatus, establish the pH conditions conducive to translocation, and establish a role in intracellular replication for the individual effectors.

## RESULTS

### *In silico* analysis.

*Edwardsiella ictaluri* encodes at least nine putative effectors with homology to effectors in other pathogens (Table 1). The previously reported leucine-rich repeat (LRR) effector EseH is on the *E. ictaluri* native plasmid pEI (16). The four LLR effectors reported here, EseJ, EseK, EseL, and EseM, were identified by Basic Local Alignment Search Tool (BLAST) analysis of the *E. ictaluri* genome using the EseH sequence as the query. All four effectors are encoded in the *E. ictaluri* chromosome but not within the T3SS PAI. Domain analyses of the five *E. ictaluri* LRR effectors indicated that the amino termini contain a translocation domain which has 53.5 to 67.7% amino acid identity (I) to, 79.4 to 88.0% amino acid similarity (S) to, and 100% query coverage (QC) of the translocation domain reported for the LRR effector SspH2 in *Salmonella* (21). The *E. ictaluri* translocation domains for the five LRR effectors have 51.9 to 82.5% I and 79.4 to 93.2% S to each other. Comparison of the central LRR regions showed that EseH contains 5 leucine-rich repeats in the core of the protein, while EseJ, EseK, EseL, and EseM have 12, 18, 6, and 11 repeats, respectively. All five of the *E. ictaluri* LRR effectors contain an E3 ubiquitin ligase domain in their carboxy terminus.

**TABLE 1:** Putative T3SS effectors identified for *Edwardsiella ictaluri*

Effector	GenBank accession no.	Size (aa)	Putative activity/description (reference)^a^
EseG	ABC60070	301	Vacuolar localization (5)
EseH	AAF85955	619	LRR, 5 repeats, E3 ubiquitin ligase (16)
EseI	AGF34188	151	*Shigella* OspB family of T3SS effectors (16)
EseJ	ACR69526	599	LRR, 12 repeats, E3 ubiquitin ligase
EseK	Genome^b^		LRR, 18 repeats, E3 ubiquitin ligase
EseL	ACR68523	705	LRR, 6 repeats, E3 ubiquitin ligase
EseM	ACR68525	793	LRR, 11 repeats, E3 ubiquitin ligase
EseN	ACR69124	214	Phosphothreonine lyase domain
EseO	ACR69900	667	*Shigella* enterotoxin 2 and ankyrin repeat protein

a LRR, leucine-rich repeat protein.

b EseK was identified during *E. ictaluri* genome sequencing but was not found in the final assembly. Analysis by PCR confirmed its presence in the genome, and it was cloned using the sequence from the original contig.

The previously described effector encoded on the *E. ictaluri* native plasmid pEI2 (5), EseI, has 30 to 50% I and 45 to 66% S to a family of hypothetical/putative proteins with similarity to OspB in *Shigella*. QC ranged from 26 to 90%, and protein analysis indicated no conserved domains or repeats. Two other *E. ictaluri* effectors with homology to those in *Shigella* were identified during annotation of the *E. ictaluri* genome. EseN has similarity to a family of T3SS effectors that carry a phosphothreonine lyase domain. EseO has homology to a family of proteins with similarity to OspD3 in *Salmonella* and carries the amino terminus of the ShET2 enterotoxin domain; with that family, it shows 30 to 77% I, 60 to 80% S, 37 to 99% QC, and an ankyrin repeat (ANK) domain. Finally, the only *E. ictaluri* effector encoded on the T3SS PAI is EseG, with 25 to 71% I to, 52 to 79% S to, 34 to 94% QC of the PRK15357 superfamily of T3SS effectors like SseG in *Salmonella*.

### *In vitro* protein secretion following a pH shift from 5 to 7.

**FIG 1:**
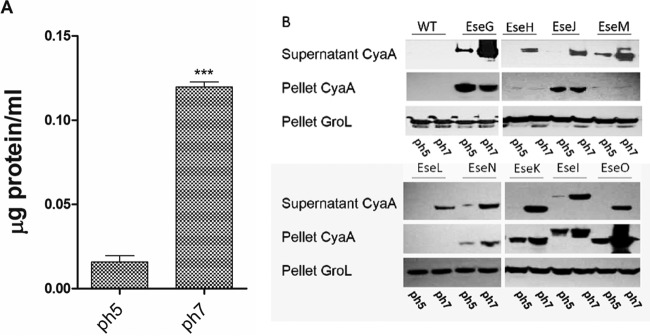
(A) Total protein secreted when *E. ictaluri* was grown overnight in MMP, pH 5, and moved to fresh MMP, pH 5. After 4 h, one tube was adjusted to pH 7, and both tubes were incubated for an additional 90 min, after which the supernatant and cell pellet fractions were separated by centrifugation. (B) Immunoblot of the supernatant and cell pellet fractions with anti-CyaA to detect the *E. ictaluri* T3SS effector-CyaA fusions. WT, wild type.

As indicated in Fig. 1A, the shift from pH 5 to pH 7 in MMP increased total protein secretion by *E. ictaluri* 10-fold compared to that in the culture kept at pH 5. As confirmation that this increase is due to increased T3SS effector release, the *E. ictaluri* effector-CyaA strains all showed an increase in release following a shift from pH 5 to pH 7 when examined in Western blots using CyaA antibody, although EseG, EseM, EseN, and EseI were also released at pH 5 in reduced quantities (Fig. 1B). EseH, EseM, and EseL were substantially absent in the pellet at either pH, suggesting that they were secreted as they were being translated. Effector secretion was not detected in the *E. ictaluri* strain that did not harbor a CyaA fusion.

### Translocation assay.

**FIG 2:**
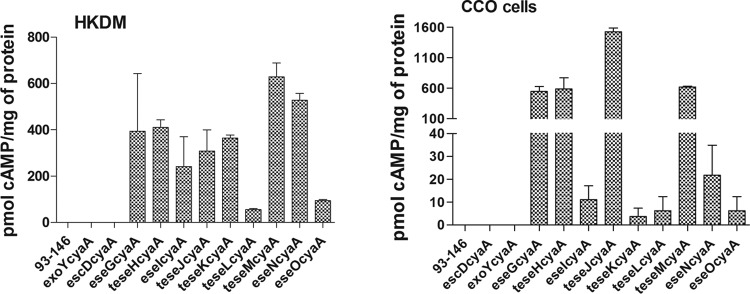
Translocation of the *E. ictaluri* effector-CyaA fusions as indicated by cAMP production in HKDM and CCO cells 7 h postinfection. Fusions for the nontranslocated *escD* and *exoY* genes were zero despite the presence of the fused AC domain of *cyaA*. The T3SS mutants carrying the effector-CyaA fusions were all negative for cAMP production (data not shown), indicating that translocation is a T3SS-dependent event. Effectors whose designations are preceded with a “t” are the leucine-rich repeat effectors that were truncated to leave only the translocation domain.

Based on the production of cAMP in the translocation assay, all *E. ictaluri* effector-CyaA fusions were translocated to the host cell cytoplasm at 7 h postinfection (p.i.) in both HKDM and CCO cells (Fig. 2). No cAMP production was detected in HKDM infected with the wild-type *E. ictaluri* strain or the T3SS knockout strain carrying the effector-CyaA constructs (data not shown). Truncation of the LRR effectors to the first 200 to 267 amino acids (aa) did not abolish translocation and resulted in greater translocation levels than in a construct carrying the entire gene (data not shown), confirming that the amino-terminal region contains the sequence responsible for translocation. The negative-control fusions for EscD and ExoY were negative for cAMP production despite the presence of the AC domain of CyaA.

### Effector translocation requires acidification and subsequent neutralization of the ECV.

**FIG 3:**
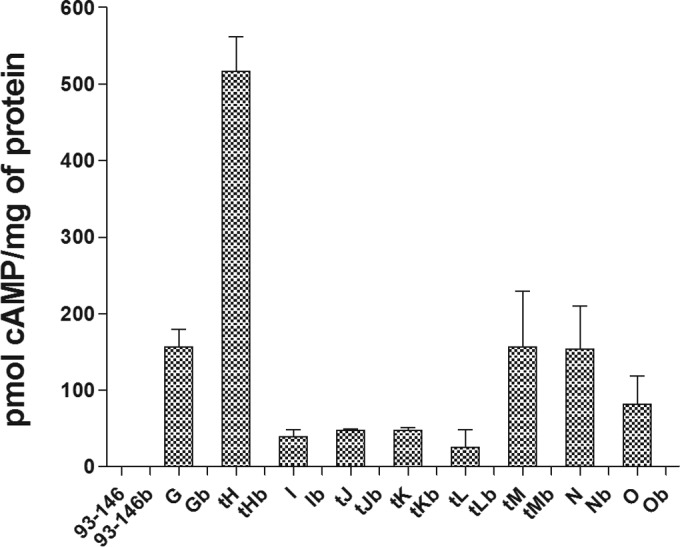
Inhibition of the vacuolar (H^+^) ATPases of HKDM by the specific inhibitor bafilomycin A_1_ to prevent acidification of the ECV totally inhibits the translocation of the *E. ictaluri* T3SS effector-CyaA fusions, as indicated by the lack of cAMP production in treated HKDM at 5 h postinfection compared to that in untreated cultures. The designations for effectors are abbreviated to just their loci. Effectors whose designations are preceded with a “t” are the leucine-rich repeat effectors that were truncated to leave only the translocation domain, and those whose designations end with a “b” were treated with bafilomycin A_1_.

**FIG 4:**
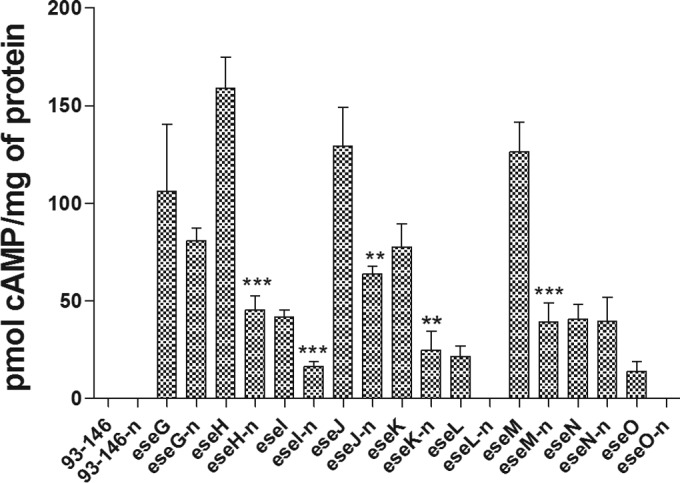
Inhibition of the HKDM-encoded arginase enzyme by the specific inhibitor norvaline to prevent neutralization of the acidified ECV inhibits the translocation of most of the effectors, as indicated by reduced cAMP production in HKDM at 5 h postinfection. Results are presented as means and standard errors of the means and are combined data from three identical experiments with two replications per treatment per experiment. Asterisks indicate a significant difference from the nontreated controls (**, *P* ≤ 0.01; ***, *P* ≤ 0.001). *P* values for Ln and On could not be calculated because all of the values were 0. Effectors whose abbreviations are preceded with a “t” are the leucine-rich repeat effectors that were truncated to leave only the translocation domain, and those whose abbreviations end with an “n” were treated with norvaline.

Treatment of HKDM with the specific vacuolar proton pump inhibitor bafilomycin A_1_ to block acidification of the ECV resulted in a complete loss of fusion protein translocation at 5 h p.i. (Fig. 3). Treatment of the cultures with the specific arginase inhibitor norvaline also resulted in a significant reduction in translocation for all effector-CyaA fusions except for EseG and EseN at 5 h p.i. (Fig. 4). This indicates that both acidification and subsequent neutralization of the ECV are required for the translocation of all of the effectors except EseG and EseN.

### Replication in channel catfish macrophages and channel catfish ovary cells.

**FIG 5:**
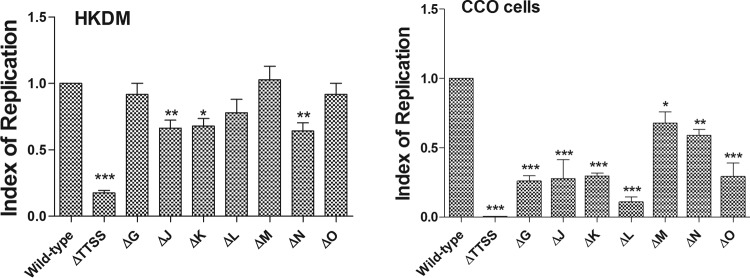
Replication of *Edwardsiella ictaluri* strains carrying mutations in T3SS effector genes in HKDM and CCO cells at 10 h postinfection. Bars indicate relative indexes of replication, which were calculated by dividing the number of CFU in the wild type and the individual mutants by the number of CFU present in the wild type. Results are presented as means and standard errors of the means and are combined data from three identical experiments, with 3 replications per treatment per experiment (*, *P* ≤ 0.1; **, *P* ≤ 0.01; ***, *P* ≤ 0.001). Complementation of EseG and EseJ in CCO cells was relatively low, at 17 and 15%, while those of EseK, EseL, EseM, EseN, and EseO were 21, 36, 22, 35, and 24%, respectively. All three of the effectors with attenuated replication in HKDM, i.e., EseJ, EseK, and EseN, were returned to wild-type levels of replication in complemented strains.

As indicated in Fig. 5, single-gene mutations of each individual effector had differential effects on intracellular replication. Only EseJ, EseK, and EseN had a significant reduction in replication in HKDM. In the nonphagocytic CCO cell line, however, all seven tested effector mutants replicated at a significantly lower rate than the wild type. Complementation of EseG and EseJ in CCO cells was relatively low at 17 and 15%, while that of EseK, EseL, EseM, EseN, and EseO was 21, 36, 22, 35, and 24%, respectively. All three of the effectors with attenuated replication in the HKDM, EseJ, EseK, and EseN, were returned to wild-type levels of replication in complemented strains. All seven had significantly greater replication than 65ST, which has a mutation in *eseU*, which encodes a major protein of the injectisome and is unable to translocate effector proteins out of the bacterial cell, as demonstrated in Fig. 2.

## DISCUSSION

The primary objectives of this study were to conduct an *in silico* analysis to identify and characterize the structures of the T3SS effectors of *E. ictaluri*, to evaluate conditions for their active translocation from the bacterium in the ECV to the host cell cytoplasm, and to establish their role in intracellular replication. The *in silico* analyses identified six new putative *E. ictaluri* T3SS effectors, bringing the total to nine. Five of the nine identified effectors, namely, EseH, EseJ, EseK, EseL, and EseM, have a translocation domain in the amino terminus, carry leucine-rich repeats (LRRs) in the central portion of the protein, and have E3 ubiquitin ligase domains in their carboxyl terminus. Leucine-rich repeats are widespread structural motifs that are found in thousands of protein sequences in all life forms, from viruses to eukaryotes (22). Despite having a wide range of functions (22, 23), LRR proteins share a structural framework, consisting of a curved solenoid structure encompassing the LRR sequence, that presents an ideal structure for ligand binding (24–26). Proteins in the LRR family form tight associations with their respective protein ligands, and the specific shape of the particular curved solenoid, as determined by the sequences of the repeats and the number of repeats, determines the protein specificity. The various lengths of the *E. ictaluri* LRR regions indicate that their solenoid structures differ, suggesting diverse types of protein binding.

As stated above, the five *E. ictaluri* LRR effectors also carry E3 ubiquitin ligase domains*.* Ubiquitin is a highly conserved 76-aa protein that controls almost all aspects of a cell’s life and death through a process known as ubiquitination, a process involving reversible covalent modifications of cellular proteins (27, 28) that is similar to phosphorylation. Ubiquitination consists of the covalent attachment of ubiquitin to lysine residues on a target protein by ubiquitin ligases (29). The number and locations of the attached moieties determine whether the protein is targeted for degradation by the proteosome or functions as a nonproteolytic signal for DNA repair, signal transduction, or vesicular trafficking (30–34). The presence of ubiquitin ligase domains on the five *E. ictaluri* LRR effectors predicts that they play an important role in determining the fate of the protein bound in the solenoid structure.

The chromosomal effector EseN, one of the four non-LRR effectors, has high homology to the OspF family of T3SS effectors, which includes OspF in *Shigella*, SpvC in *Salmonella*, and HopA11 in *Pseudomonas* (35), as well as 95 others identified in a BLAST search of microbial genomes. Members of the OspF family act as phosphothreonine lyases (PTL), which catalyze the removal of the phosphate group of phosphothreonine in the pT-X-pY motif of phosphorylated mitogen-activated protein kinases (MAPK), preventing downstream phosphorylation of histone H3 and downregulating transcription of proinflammatory cytokines, resulting in attenuation of the host inflammatory response (36). EseN also carries the GDKXH motif, which is required for PTL activity (35), as well as the highly conserved D motif for MAPK substrate docking in the amino terminus (35), suggesting a function for EseN similar to those of the other members of the family.

EseO, a second non-LRR effector, is also located on the *E. ictaluri* chromosome, and BLAST analysis identified two conserved domains, *Shigella* enterotoxin 2 (ShET2) and ankyrin repeats (ANKs). ShET2 is a widespread domain in *Shigella* and *Escherichia coli* that is responsible for causing diarrhea (37, 38) but also for upregulating interleukin 8 (IL-8) secretion in epithelial cells in *Shigella* (39). Ankyrin repeats are common protein-protein interaction motifs in a wide variety of eukaryotic and prokaryotic proteins, including a diverse family of type IV secretion system effectors (40). This is the first report of an ANK protein being translocated by a T3SS.

Both SseG and SseF are encoded on the chromosome in the *Salmonella* pathogenicity island 2 (SPI-2) T3SS PAI of *Salmonella*. In epithelial cells, SseG and SseF are involved in placement of the developing *Salmonella*-containing vacuoles (SCVs) adjacent to the nucleus, locating in the region of the microtubule-organizing center and associated Golgi stacks, a location that is required for *Salmonella* replication (41, 42). Single SseG and SseF mutants have similar levels of attenuation in mice and growth attenuation in macrophages (43, 44), but SseG SseF double mutants remain as virulent as the single mutants (45). The similarity of the virulence of the single mutants and the lack of an additive effect on virulence in the double mutant, along with the 30% similarity of the amino acid sequences, suggest that SseG and SseF have redundant functions and that *E. ictaluri* EseG alone is sufficient to achieve the same result as SseF SseG in *Salmonella*. *Edwardsiella tarda* EseG triggers microtubule destabilization in human embryonic kidney 293 (HEK293A) cells, but positioning of the vacuole was not evaluated (46).

Based on immunofluorescence and cell fractionation assays in the zebrafish ZF4 fibroblast cell line, *E. ictaluri* EseI appeared to localize to the cytosolic fraction when it was expressed in *E. tarda*. Adhesion studies of epithelioma papulosum cyprini (EPC) carp cells further suggested that EseI was involved in adhesion and invasion of that cell type (47). Expression of *E. ictaluri* EseI in the surrogate *E. tarda* makes it difficult to interpret these conclusions regarding the function of EseI in light of the data that suggest that the *Shigella* homologue of EseI, OspB, functions as an immunomodulator (48, 49), not an adhesion molecule.

The T3SS-encoded regulatory proteins EsrABC upregulate expression of the components of the T3SS that are encoded on of the PAI in response to low pH and low phosphate in minimal media (50). Plasmid-encoded EseI, however, is also upregulated by low pH and low phosphate in minimal media but not in an EsrABC-dependent manner (50). EseI was also secreted when it was expressed in *E. tarda* but not in a T3SS-dependent manner (47). Although these data suggest that EseI secretion in media is not linked to the T3SS, the *ex vivo* data presented here showing that EseI is translocated in wild-type *E. ictaluri* but not in the T3SS knockout mutant 65ST indicates that the T3SS is required for translocation in *E. ictaluri* HKDM and the CCO cell line. Zhao et al. (47) also demonstrated that EseI was translocated in the mammalian J774 macrophage cell line but did not report data for translocation in a T3SS mutant.

Baumgartner et al. (13) previously demonstrated that the development of the ECV involves initial acidification of the ECV by vacuolar H^+^ ATPases, which triggers expression of the T3SS (50) and activates the *E. ictaluri* acid-activated urease (15). Activation of the urease results in the production of ammonia by means of urea produced by the HKDM-encoded arginase enzyme, which results in an increase in the pH of the ECV (13). Results presented here demonstrate that both acidification and neutralization of the ECV are required to trigger the translocation of the *E. ictaluri* effectors, which is unique among bacterial pathogens. As with the *E. ictaluri* T3SS, transcription and assembly of the related *Salmonella* SPI-2 T3SS requires acidification of the SCV, but the SCV remains acidified. The signal for translocation, however, is also recognition of a neutral pH, but the signal recognized is the neutral pH of the cytoplasm by the effectors SsaM, SpiC, and SsaL (51), which do not have homologues in the *E. ictaluri* genome.

Attenuation of intracellular replication of the individual effector mutants is most pronounced in the CCO cell line, with all seven effector mutants demonstrating significant reductions in intracellular growth. This is in contrast to the impact in HKDM, in which only EseJ, EseK, and EseN were significantly attenuated, and the level of attenuation was less than in the CCO cells. The negligible effect of only three of the single-effector mutants in HKDM, compared to the severe replication defect of an apparatus mutant, 65ST, which precludes the translocation of any effectors, may suggest the presence of additional effectors in the *E. ictaluri* genome that have a role in intracellular replication in HKDM. The differential levels of attenuation for intracellular replication between HKDM and CCO cells may indicate differential modes of action in the 2 cell types. Further work to establish the biochemical and physiological activities of the individual effectors in both HKDM and CCO cells is required.

## MATERIALS AND METHODS

### *In silico* analysis.

BLAST was used to align the three putative effectors previously identified for *E. ictaluri* to the genomic sequence of *E. ictaluri* (GenBank accession number CP001600) in order to identify additional effectors. ClustalX was used for multiple-sequence alignments of DNA and protein sequences. The InterPro database (http://www.ebi.ac.uk/Tools/pfa/iprscan/) was used for possible protein family identification and to identify conserved domains within the protein sequence.

### Bacterial strains, plasmids, and media.

Strains and plasmids used in this study are listed in Table 2. *Edwardsiella ictaluri* strains were grown at 28°C on either Trypticase soy agar plates supplemented with 5% sheep blood (BA; Remel Products, Lenexa, KS) or porcine brain heart infusion (BHI) agar (BD Difco, Lawrence, KS). Broth cultures of *E. ictaluri* were grown in either porcine BHI broth or *E. ictaluri* low-phosphate minimal medium (MMP) (50, 52). *Escherichia coli* strains were cultured using LB broth or agar at 37°C (BD Difco). All cultures grown in broth were aerated on a Cel-Gro tissue culture rotator (Lab-Line, Inc., Melrose Park, IL). Antibiotics were added where appropriate in the following concentrations: for ampicillin (Amp), 200 µg/ml, and for colistin (Col), 20 µg/ml (Sigma).

**TABLE 2:** Bacterial strains and plasmids used in this study

Strain or plasmid	Relevant characteristic(s)^a^	Reference or source
Strains		
*E. coli* S17 λ-*pir*	(F^−^) RP4-2-Tc::Mu *aphA*::Tn*7 recA* λ-*pir*	61
* E. ictaluri*		
93-146	Wild-type *E. ictaluri* isolated from a moribund channel catfish from a natural outbreak at a commercial facility in 1993	LSU aquatic diagnostic laboratory
65ST	93-146 *esaU*::Tn*5*Km2	5
93-146 *eseG*::*cyaA*	Carrying pBBR1, *eseG*::*cyaA* Amp^r^	This work
Δ65 ST *eseG*::*cyaA*	Carrying pBBR1, *eseG*::*cyaA* Amp^r^	This work
93-146 *teseH*::*cyaA*	Carrying pBBR1, truncated *eseH*::*cyaA*, Amp^r^	This work
Δ65 ST *teseH*::*cyaA*	Carrying pBBR1, truncated *eseH*::*cyaA*, Amp^r^	This work
93-146 *eseI*::*cyaA*	Carrying pBBR1, *eseI*::*cyaA* Amp^r^	This work
Δ65 ST *eseI*::*cyaA*	Carrying pBBR1, *eseI*::*cyaA* Amp^r^	This work
93-146 *teseJ*::*cyaA*	Carrying pBBR1, truncated *eseJ*::*cyaA*, Amp^r^	This work
Δ65 ST *teseJ*::*cyaA*	Carrying pBBR1, truncated *eseJ*::*cyaA*, Amp^r^	This work
93-146 *teseK*::*cyaA*	Carrying pBBR1, truncated *eseK*::*cyaA*, Amp^r^	This work
Δ65 ST *teseK*::*cyaA*	Carrying pBBR1, truncated *eseK*::*cyaA*, Amp^r^	This work
93-146 *teseL*::*cyaA*	Carrying pBBR1, truncated *eseL*::*cyaA*, Amp^r^	This work
Δ65 ST *teseL*::*cyaA*	Carrying pBBR1, truncated *eseL*::*cyaA*, Amp^r^	This work
93-146 *teseM*::*cyaA*	Carrying pBBR1, truncated *eseM*::*cyaA*, Amp^r^	This work
Δ65 ST *teseM*::*cyaA*	Carrying pBBR1, truncated *eseM*::*cyaA*, Amp^r^	This work
93-146 *eseN*::*cyaA*	Carrying pBBR1, *eseN*::*cyaA* Amp^r^	This work
Δ65 ST *eseN*::*cyaA*	Carrying pBBR1, *eseN*::*cyaA* Amp^r^	This work
93-146 *eseO*::*cyaA*	Carrying pBBR1, *eseO*::*cyaA* Amp^r^	This work
Δ65 ST *eseO*::*cyaA*	Carrying pBBR1, *eseO*::*cyaA* Amp^r^	This work
ΔG	*E. ictaluri* 93-146 Δ(*eseG*_1–773_)	This work
ΔJ	*E. ictaluri* 93-146 Δ(*eseJ*)	This work
ΔK	*E. ictaluri* 93-146 Δ(*eseK*_1–2202_)	This work
ΔL	*E. ictaluri* 93-146 Δ(*eseL*_82–2094_)	This work
ΔM	*E. ictaluri* 93-146 Δ(*eseM*)	This work
ΔN	*E. ictaluri* 93-146 Δ(*eseN*)	This work
ΔO	*E. ictaluri* 93-146 Δ(*eseO*_1–1873_)	This work
Plasmids		
pEI1	*E. ictaluri* 93-146 native plasmid	16
pEI2	*E. ictaluri* 93-146 native plasmid	16
pMJH20	Plasmid containing CyaA adenylate cyclase	17
pBBR1MCS-4	Broad-host-range expression vector	54
pBBR1-*eseG*::*cyaA*	pBBR1MCS4 carrying *eseG*::*cyaA*	This work
pBBR1-*eseI*::*cyaA*	pBBR1MCS4 carrying *eseI*::*cyaA*	This work
pBBR1-*eseO*::*cyaA*	pBBR1MCS4 carrying *eseO*::*cyaA*	This work
pBBR1-*eseN*::*cyaA*	pBBR1MCS4 carrying *eseN*::*cyaA*	This work
pBBR1-*eseG*::*cyaA*	pBBR1MCS4 carrying *eseG*::*cyaA*	This work
pBBR1-t*eseH*::*cyaA*	pBBR1MCS4 carrying truncated *eseH*::*cyaA*	This work
pBBR1-*eseI*::*cyaA*	pBBR1MCS4 carrying *eseI*::*cyaA*	This work
pBBR1-t*eseJ*::*cyaA*	pBBR1MCS4 carrying truncated *eseJ*::*cyaA*	This work
pBBR1-t*eseK*::*cyaA*	pBBR1MCS4 carrying truncated *eseK*::*cyaA*	This work
pBBR1-t*eseL*::*cyaA*	pBBR1MCS4 carrying truncated *eseL*::*cyaA*	This work
pBBR1-t*eseM*::*cyaA*	pBBR1MCS4 carrying truncated *eseM*::*cyaA*	This work
pRE107-Δ*eseG*	pRR107 with individual effector deletion	This work
pRE107-Δ*eseJ*	pRR107 with individual effector deletion	This work
pRE107-Δ*eseK*	pRR107 with individual effector deletion	This work
pRE107-Δ*eseL*	pRR107 with individual effector deletion	This work
pRE107-Δ*eseM*	pRR107 with individual effector deletion	This work
pRE107-Δ*eseN*	pRR107 with individual effector deletion	This work
pRE107-Δ*eseO*	pRR107 with individual effector deletion	This work
pBBR1-*eseG*	Complementation plasmid	This work
pBBR1-*eseJ*	Complementation plasmid	This work
pBBR1-*eseK*	Complementation plasmid	This work
pBBR1-*eseL*	Complementation plasmid	This work
pBBR1-*eseM*	Complementation plasmid	This work
pBBR1-*eseN*	Complementation plasmid	This work
pBBR1-*eseO*	Complementation plasmid	This work

a A subscript number range after a gene name indicates the range of base pairs left in the gene after deletion.

### SPF channel catfish.

Channel catfish egg masses obtained from production facilities with no history of *E. ictaluri* outbreaks were disinfected with 100 ppm free iodine and were hatched and reared in closed recirculating aquaculture systems in the specific-pathogen-free (SPF) aquatic laboratory at the LSU School of Veterinary Medicine. Catfish used for harvesting macrophages were reared in the SPF lab and were between 500 and 750 g. All animal work was conducted under protocols approved by the Institutional Animal Care and Use Committee.

### DNA manipulations.

DNA manipulations were performed by standard methods. All enzymes used in plasmid construction were obtained from New England Biolabs (Beverly, MA). Total DNA was purified from cultures using the High Pure PCR template preparation kit (Roche, Mannheim, Germany). Phusion high-fidelity polymerase (Thermo Scientific, Waltham, MA) was used for PCR amplifications. DNA restriction fragment isolation and PCR product purification were done by using the QIAquick gel extraction kit (Qiagen, Valencia, CA). All procedures were performed according to the manufacturer’s instructions. Oligonucleotide primers were purchased from Integrated DNA Technologies (Coralville, IA). All constructs were confirmed by PCR and DNA sequencing. All DNA work was conducted under protocols approved by the Inter-Institutional Biological and Recombinant DNA Safety Committee of Louisiana State University.

### Effector-CyaA fusion and effector mutant construction.

**FIG 6:**
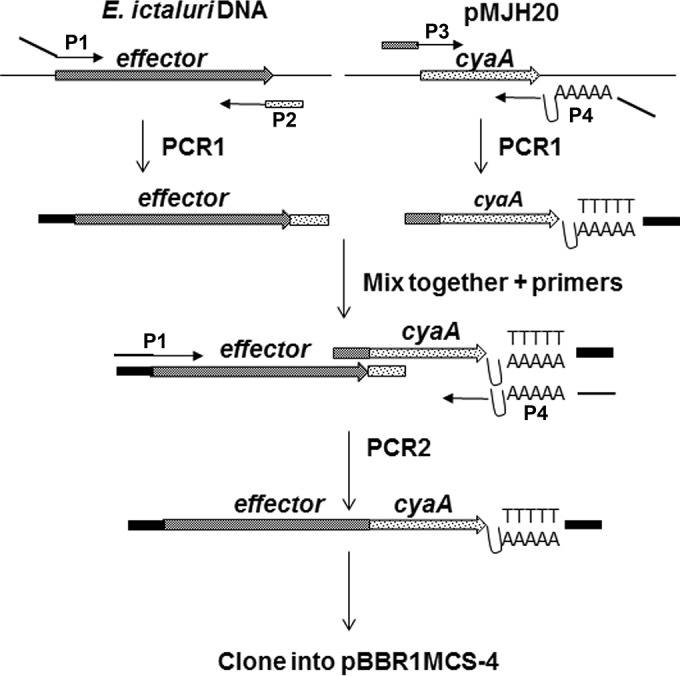
Schematic describing the construction of the *E. ictaluri* effector mutants. The LRR effector constructs were made by truncating the protein to eliminate the LRR region and the carboxy terminus.

The strategy for constructing the effector-CyaA fusions is depicted in Fig. 6. Briefly, each effector was amplified with a gene-specific forward primer, P1, that annealed at least 300 bp upstream from the translational start codon in order to incorporate the native promoter and included a linker containing a specific restriction enzyme (RE) site to facilitate cloning. Primer P1 was paired with the reverse primer, P2, which included a linker that contained 25 to 30 bp of the AC domain of CyaA. The 1,197-bp AC domain of CyaA from bp +4 to +1227 was amplified from the plasmid pMJH20 (17) with primer P3, which included 25 to 30 bp of the specific effector, and primer P4, which included an in-frame stop codon and the rho-independent transcriptional terminator from the *Bacillus subtilis* yqfT gene (53) and another RE site. Both *eseI* and *eseG* were amplified to include their respective chaperones, *escD* and *escB.* Because fusion of CyaA to the intact effectors carrying leucine-rich repeats (LRRs) resulted in poor translocation, the fusions for the five LRR proteins were constructed with reverse primers that amplified approximately 200 amino acids of the amino terminus, removing the LRR region and the carboxy terminus from the fusion construct. To serve as negative controls, CyaA fusions were also constructed for the chaperone for EseH, namely, EscD, and the *E. ictaluri* adenylate cyclase ExoY, neither of which should be translocated. Primers used to amplify the AC domain of CyaA and the individual effectors are listed in Table 3. To produce the fusion constructs, the effector and CyaA amplicons were mixed and amplified using primers P1 and P4, and the construct was cloned into the plasmid pBBR1MCS-4 (54). The resulting plasmids were transformed into *E. coli* S17-1 λ-pir (55) and transferred to *E. ictaluri* by conjugation (56).

**TABLE 3:** Primers used to construct the effector-*cyaA* fusions in this study^a^

Primer	Enzyme	Sequence 5′–3′
eseG P1	XbaI	GTACGCTCGAG**TCTAGA**TCGTCTAGAATCGGGCGCTGGATAAGATGCGACGACGCCTGAC
eseG P2		GCGTAACCAGCCTGATGCGATTGCTGAAAGAAGCATGCGGCAAAGCTGTGGCGTCGTGTC
eseG P3		GACACGACGCCACAGCTTTGCCGCATGCTTCTTTCAGCAATCGCATCAGGCTGGTTACGC
eseI P1	SacI	GCCGAT**GAGCTC**TGGCTCCCTAATCCTGTCTTGCGGCGAGCGGTGGGGATGAATGCAACG
eseI P2		CGTTTGCGTAACCAGCCTGATGCGATTGCTGGAAGAAGCATGCGGCTGGGATGAAGACTC
eseI P3		GAGTCTTCATCCCAGCCGCATGCTTCTTCCAGCAATCGCATCAGGCTGGTTACGCAAACG
eseH P1	SacI	GCGACT**GAGCTC**AGCCATTCACGACACTGCATGACGGTCAGATTCAGC
treseH P2		CGTAACCAGCCTGATGCGATTGCTGTTTGGTGTTAGAGACATCCAGCCGGGTGTTATATC
eseH P3		ACACCCGGCTGGATGTCTCTAACACCAAACAGCAATCGCATCAGGCTGGTTACGC
eseJ P1	SacI	GCCGAT**GAGCTC**TCTAAATAGCAGCAGGTTCAGAGGAGTAAC
treseJ P2		CGTTTGCGTAACCAGCCTGATGCGATTGCTGGCAGTGACGTAGCTTCCACACAG
eseJ P3		TACTGTGTGGAAGCTACGTCACTGCCAGCAATCGCATCAGGCTGGTTACGCAAACG
eseK P1	SacI	GCCGAT**GAGCTC**GTGATCTACACAACGAATGCTATCGAGT
treseK P2		GGCGGCGTTTGCGTAACCAGCCTGATGCGATTGCTGCAGTCCGGTGGGCAGCGGCGGCAG
eseK P3		CTGCCGCCGCTGCCCACCGGACTGCAGCAATCGCATCAGGCTGGTTACGCAAACGCCGCC
eseL P1	SacI	GCCGAT**GAGCTC**ATAGCGTAGGGTGTCGATGCTACAGCCGATC
treseL P2		GTTTGCGTAACCAGCCTGATGCGATTGCTGGAGAGAGACATTCAGCCACTGCAGTCCG
eseL P3		AACGGACTGCAGTGGCTGAATGTCTCTCTCCAGCAATCGCATCAGGCTGGTTACGCAAAC
eseM P1	SacI	GTACGCTC**GAGTCT**CTAGAAAATGGTCTGTACAACGCGGAGGTGATACACCGACAG
treseM P2		TTTGCGTAACCAGCCTGATGCGATTGCTGATGCATGCAGCGACGTAGCCGCAGCACTG
eseM P3		CAGTGCTGCGGCTACGTCGCTGCATGCATCAGCAATCGCATCAGGCTGGTTACGCAAA
eseO P1	KpnI	GCCGAT**GGTACC**CGGATCCTTTGTCATATCATTGTCTTCCCTCCTG
eseO P2		GTTTGCGTAACCAGCCTGATGCGATTGCTGTGTATGATTAGGGTCGTAGAGGTAAATCAC
eseO P3		GTGATTTACCTCTACGACCCTAATCATACACAGCAATCGCATCAGGCTGGTTACGCAAAC
eseN P1	KpnI	GACGCTCGA**GGTACC**GTCTAGAAAGTTGAGCTGGAAGGTTTTCAGG
eseN P2		GCGTTTGCGTAACCAGCCTGATGCGATTGCTGCTCTGTCATTAAACGATAAAACGGCTCC
eseN P3		GGAGCCGTTTTATCGTTTAATGACAGAGCAGCAATCGCATCAGGCTGGTTACGCAAACGC
exoY P1	SacI	GCCGAT**GAGCTC**GATATCAAGCTGGTTGCGGATACACGCGATG
exoY P2		CGTTTGCGTAACCAGCCTGATGCGATTGCTGTCTCGGTTTTGTTAACGGATC
exoY P3		TTAAATTAGATCCGTTAACAAAACCGAGACAGCAATCGCATCAGGCTGGTTACGCAAACG
P4	XbaI	GAGCGTACC**TCTAG*A****AAAAATGGGGGATAACACCCCCATT*ATTGGCGTTCCACTGCGCCCAGCGACGGCCGCCGCCGCAATCCGGGTG

a Primers starting with “tr” indicate an LRR effector that is truncated by removal of the LRR and the carboxy terminus. Final letters in the primer name indicate the primers identified in Fig. 6. Underlined sequences indicate the *cyaA* overlap. Bold sequences indicate the restriction enzyme named in the second column. Italics in the P4 primer indicate the *rho*-independent transcriptional terminator from the *B. subtilis* yqfT gene (53).

Individual effector mutants were constructed in a similar manner except that primers P1 and P2 amplified from the amino terminus to bp 500 to 900 of the flanking sequence of the effector and primers P3 and P4 amplified from the carboxy terminus to bp 500 to 900 of the flanking sequence. Primers P1 and P4 contained selected restriction endonucleases to facilitate final cloning of the gene-deleted fragment. Primer P2 included overlapping sequence of the right arm, and P3 contained overlapping sequence of the left arm so that when the PCR products were mixed, they annealed to each other; amplification with the P1 and P4 primers resulted in a fragment with the required deletion and with flanking sequence on either side of the effector to be deleted to mediate integration of the plasmid into the chromosome. Primers used for the construction of the single-gene effector mutants are listed in Table 4. The deletion constructs were cloned into the suicide plasmid pRE107 (57), transformed into *E. coli* S17 λ-*pir*, transferred to *E. ictaluri* 93-146 by conjugation, and grown in BHI-Amp to select for plasmid integration into the chromosome. Colonies positive for Amp^r^ were then grown in BHI with 7.5% sucrose to select for a second crossover and excision of the plasmid, which resulted in a mixture of wild-type and deletion mutants. Deletion mutants were identified by PCR using effector-specific primers and confirmed by DNA sequencing. Final mutant constructs consisted of complete gene deletions of EseJ, EseM, and EseN, deletions of bp 1 to 773 of a total of 921 bp for EseG, bp 1 to 2202 of a total of 2,810 bp for EseK, bp 82 to 2094 of a total of 2,175 bp for EseL, and bp 1 to 1873 of a total of 1,979 bp for EseO. Previous work (5) demonstrated that introduction of mutations in *eseH* and *eseI* on the *E. ictaluri* plasmids resulted in the production of strains carrying 50% mutant plasmid constructs and 50% wild-type plasmid constructs. The presence of both mutant and wild-type constructs makes interpretation of results difficult, so new mutants of EseH and EseI were not constructed for use in this study.

**TABLE 4:** Primers used for construction of the single-gene effector mutants

Primer name	Sequence (restriction endonuclease)^a^
eseG P1	GAATCGTGTACAGG**GTCGAC**GATGGATGACGTCAGCCGTTTC (SalI)
eseG P2	TGTGGCTTCGAGCTCAGCCATCCGTTCGTCTTAAGGTTGATTAAGCGTATCCAGCAG
eseG P3	CTGCTGGATACGCTTAATCAACCTTAAGACGAACGGATGGCTGAGCTCGAA GCCACAG
eseG P4	CACGATGCC**TCTAGA**TACTGACGGTTTCACGGTTTTGTTCCTGGTTAAGA (XbaI)
eseJ P1	GGACTATCT**GAGCTC**GGGGCCAGGAAACAGGACGTAACCCGACAGAC (SacI)
eseJ P2	GCCACCGCTCACGGTTACCGCACGTAGTGAAATTTTCCCATTAATTCAGTTG
eseJ P3	CAACTGAATTAATGGGAAAATTTCACTACGTGCGGTAACCGTGAGCGGTGGC
eseJ P4	CACGATGCC**TCTAGA**AGTTAGAACTTAAAAAAACGCGGAACACATC (XbaI)
eseK P1	GGACTATCT**GAGCTC**TCTGGCTCAATGTGCTGACAGAGCTGAAG (SacI)
eseK P2	CTACCGACACGAGCCCCGGGATATCGTTAGTACAATTTTCCTATTGATTCATTGG
eseK P3	CCAATGAATCAATAGGAAAATTGTACTAACGATATCCCGGGGCTCGTGTCGGTAG
eseK P4	CACGATGCC**TCTAGA**TCAGTTTATGCCAGGGAATGCTATACAGGGGACGCATC (XbaI)
eseL P1	GAATCGTGTACAGG**GTCGAC**GAAAAAAATCTGCCGGGGTGGGTCAGGTC (SalI)
eseL P2	CGGCCACCGCTCACGGTTACCGCTCGTCTCATTTCCGGTGGGGTATTAGCGCTGGC
eseL P3	GCCAGCGCTAATACCCCACCGGAAATGAGACGAGCGGTAACCGTGAGCGGTGGCCG
eseL P4	CACGATGCC**TCTAGA**TACTGGAACGGGTCGGTCATATCCCCCCGGCTG (XbaI)
eseM P1	GAATCGTGTACAGG**GTCGAC**TCCCGAACTTCACTGTCAATCAATTTCATA (SalI)
eseM P2	CACCGCTCATGGTTACCGCACGTAGTGAAATTTTCCCATTAATTCAGTGG
eseM P3	CCACTGAATTAATGGGAAAATTTCACTACGTGCGGTAACCATGAGCGGTG
eseM P4	CACGATGCC**TCTAGA**TTTCGACTTTACGCTGATCTTTGCTGAACCGTAGCGGATTTC (XbaI)
eseN P1	GAATCGTGTACAGG**GTCGAC**TATCAGCATGGCTGCCTCTTTATAACCAGATAG (SalI)
eseN P2	CGCCTTCCGTCATCACCTCAGCGCTACGCGGGGGGCATCTTCTGCCTCCCGGCGGTAGGC
eseN P3	GCCTACCGCCGGGAGGCAGAAGATGCCCCCCGCGTAGCGCTGAGGTGATGACGGAAGGCG
eseN P4	CACGATGCC**TCTAGA**CCTGAACTTTCTGCGCCCGTGGGTTATCGAGGCCTTCGGCGAC (XbaI)
eseO P1	GGACTATCT**GAGCTC**TGCAGCTTGTTGGTCGCCAGCGCCTGGGC (SacI)
eseO P2	CATATGGAATGACGCCTGTATCGTTAATAAAATATATTAATACCTTATGTTATCCTATC
eseO P3	GATAGGATAACATAAGGTATTAATATATTTTATTAACGATACAGGCGTCATTCCATATG
eseO P4	CACGATGCC**TCTAGA**CATCACGGTCTGACCTGTCCTGCCATCACGTC (XbaI)

a Underlined sequences represent the overlapping sequences in P2 and P3 that mediate annealing of the two amplicons to enable amplification of the complete gene-deleted fragment with P1 and P4. Restriction endonuclease sites to facilitate final cloning of the gene-deleted fragment are in bold.

### *In vitro* secretion following a pH shift from 5 to 7.

The *E. ictaluri* effector-CyaA strains and the wild type were cultured for 16 to 18 h to an optical density at 600 nm (OD_600_) of 1.8 to 2.0 to achieve maximum cell density. Cells were pelleted at 5,000 rpm for 5 min, washed once in phosphate-buffered saline, and resuspended in 2 ml of MMP, pH 5. One milliliter of this suspension was inoculated into each of two 5-ml tubes of MMP, pH 5, and incubated for 4 h at 28°C, after which the pH of one culture was maintained at pH 5 while the other was adjusted to pH 7. Both were incubated an additional 90 min at 28°C, after which the cells were pelleted at 6,000 rpm for 6 min and separated into the supernatant and cell pellet fractions. Bacterial pellets were resuspend in XT sample buffer (Bio-Rad, Hercules, CA) and boiled for 5 min, after which 1× Halt protease inhibitor (Thermo Scientific) and 25 U Pierce Universal nuclease (Thermo Scientific) were added. Supernatants were concentrated to 100 µl with a Spin-X UF6 10,000-molecular-weight-cutoff concentrator (Corning, Lowell, MA), and 25 µl of XT sample buffer (Bio-Rad) was added, after which the sample was boiled for 5 min. Halt protease inhibitor (Thermo Scientific) was added to the supernatant fraction, and total protein was determined by using the Bio-Rad, Bradford protein assay.

Samples were separated by SDS-PAGE on 4 to 12% polyacrylamide gradient gels and transferred onto polyvinylidene difluoride (PVDF) membranes by using the iBlot dry transfer system (Life Technologies, Grand Island, NY). Membranes were blocked with 5% blotting-grade nonfat milk (Bio-Rad) in Tris-buffered saline with 0.1% Tween 20 (Sigma) for 1 h. Effector-CyaA fusion proteins were detected using monoclonal antibody 3D1 against CyaA (Santa Cruz Biotechnology, Dallas, TX). As a reference protein, GroL was detected using rabbit polyclonal antibody against *E. coli* GroL (Assay Designs, Ann Arbor, MI). For detection, goat anti-mouse or goat anti-rabbit streptavidin–poly-horseradish peroxidase (HRP) (Thermo Scientific) was used at a dilution of 1:10,000, followed by chemiluminescence detection using the SuperSignal West Pico chemiluminescent substrate (Thermo Scientific).

### Cell culture.

For the macrophage studies, HKDM were isolated by the method of Booth et al. (3), and viable counts were determined using trypan blue dye exclusion (58). Dissociated cells were suspended to a final concentration of 1 × 10^7^ cells/ml in channel catfish macrophage medium (CCMM), which consists of RPMI 1640 medium (Gibco, Invitrogen Corporation, Carlsbad, CA) diluted to a catfish tonicity of 243 mosmol/kg by addition of 1 part sterile deionized/distilled water (RPMI 9:1) and contains 15 mM HEPES buffer solution (Gibco), 0.18% sodium bicarbonate solution (Gibco), 0.05 mM 2-beta-mercaptoethanol (Sigma Chemicals Co., St. Louis, MO), and 5% heat-inactivated pooled channel catfish serum (58). One milliliter of the cell suspension was added to each well of a 24-well plate and allowed to adhere for 16 h at 28°C with 5% CO_2_, after which the wells were washed three times with RPMI 9:1 to remove nonadherent cells and 1 ml of fresh CCMM was added per well. To evaluate replication, 1 × 10^4^ cells of either wild-type or mutant *E. ictaluri* that had been opsonized for 30 min in normal autologous serum were added to triplicate wells of the 16-h HKDM cultures, giving a multiplicity of infection (MOI) of 1 bacterium per 10 HKDM. After infection, plates were centrifuged at 400 × *g* to synchronize the contact of the bacteria with the adhered cell layer and allowed to incubate for 30 min at 28 C with 5% CO_2_. The medium was then removed from each well, and CCMM with 100 µg/ml gentamicin was added for 1 h to kill residual extracellular bacteria. Cells were then washed three times with RPMI 9:1 and CCMM containing a 0.35-µg/ml bacteriostatic dose of gentamicin to control the extracellular growth of any bacteria released from the cells. At 0, 4, 8, or 10 h, depending on the assay, the HKDM were lysed by the addition of 100 µl of a 1% solution of Triton X-100 (Fisher Scientific, Fair Lawn, NJ) and the numbers of surviving *E. ictaluri* cells were determined by spreading serial dilutions on BA. A similar assay was done using the CCO cell line except that RPMI 1640 medium (Gibco) with 10% fetal bovine serum, 25 mM HEPES, and 0.01 mM sodium pyruvate was used, cells were split and passaged using standard cell culture methods, and the MOI was 1 bacterium per cell.

### Translocation assay.

Translocation of the effector-CyaA fusions from *E. ictaluri* in the ECV to the cytoplasm was determined using both HKDM and CCO cells by measuring cAMP production. Briefly, duplicate wells of HKDM were infected at an MOI of 10 bacteria to 1 HKDM, and CCO cells were infected at an MOI of 100:1. Following infection, any remaining extracellular bacteria were killed using 100 µg/ml of gentamicin. At 7 h postinfection, cells were lysed with 0.1 M HCl containing 0.1% Triton X-100. Levels of cAMP produced by the interaction of the AC domain of the CyaA toxin with calmodulin in the host cell cytoplasm were measured in picomoles of cAMP per milliliter by using the cAMP complete enzyme-linked immunosorbent assay (ELISA) kit from Enzo Life Sciences (Plymouth Meeting, PA). Production of cAMP was normalized by determining the protein concentration in each sample using the Bio-Rad protein assay and calculating the number of picomoles of cAMP per milligram of protein.

### Effect of vacuolar pH on translocation.

To evaluate the requirement for initial acidification of the ECV, the translocation assay was done with all *E. ictaluri* effector-CyaA strains in HKDM cultured with a 10 nM concentration of the specific inhibitor of vacuolar H^+^ ATPases throughout the assay, bafilomycin A_1_ (59). Untreated HKDM were used as a control. Cells were lysed 5 h p.i. and assayed for cAMP production.

To prevent subsequent neutralization of the ECV, the translocation assay was also done with all *E. ictaluri* effector-CyaA strains using HKDM cultured throughout the assay with a 10 mM concentration of the specific arginase inhibitor norvaline. Untreated HKDM were used as a positive control. Cells were lysed 5 h p.i. and assayed for cAMP production.

### Replication in channel catfish macrophages and channel catfish ovary cells.

Head kidney-derived macrophages were collected from channel catfish, and CCO cells were maintained using standard cell culture techniques. For complementation, individual effectors and their promoter regions were amplified by PCR using the primers in Table 5 and cloned into the stably expressed complementation plasmid PBBR1MCS-4 (54, 60), and the fidelity of the amplified products was confirmed by DNA sequencing. Both cultures were infected with wild-type and mutant *E. ictaluri* strains, as well as the strains carrying the corresponding complementation plasmid, and evaluated for replication using the gentamicin exclusion assay, with 3 to 4 replicate wells per treatment. Cell lysates were serially diluted after 10 h and plated on BA plates, and numbers of CFU per well were determined. An index of replication was calculated by dividing the number of CFU present in the individual mutants by the number of CFU present in the wild type. Gentamicin exclusion experiments were repeated 3 to 4 times to establish reproducibility.

**TABLE 5:** Primers used for construction of the complementation plasmids

Primer	Enzyme	Sequence 5′–3′^a^
eseG P1	XbaI	GTACGCTCGAG**TCTAGA**TCGTCTAGAATCGGGCGCTGGATAAGATGCGACGACGCCTGAC
eseG P2	SalI	GTACGCTCGAT**GCTGTC**GACTCAGGCAAAGCTGTGGCGTCGTGTCAGTGGAGCAG
eseJ P1	SacI	GCCGAT**GAGCTC**TCTAAATAGCAGCAGGTTCAGAGGAGTAAC
eseJ P2	HindIII	GCCGC**AAGCTT**CGCCAGAGAATGATATACAGAGGACTCAGCTAACGAC
eseK P1	SacI	GCCGAT**GAGCTC**GTGATCTACACAACGAATGCTATCGAGT
eseK P2	XhoI	GTACGCTCGATG**CTCGAG**TCAGTTTATGCCAGGGAATGCTATACAGGGGACGCATCTAG
eseL P1	SacI	GCCGAT**GAGCTC**ATAGCGTAGGGTGTCGATGCTACAGCCGATC
eseL P2	HindIII	GGCCGC**AAGCTT**CGCCAGAGAATGATATACAGAGGACTCAG
eseM P1	KPN	GTACGCTCGATG**GGTACC**AAATGGTCTGTACAACGCGGAGGTGATACACCGACAG
eseM P2	XbaI	GTACGCTCGATG**TCTAGA**CTCACAACTGCCGAACGTGGACTGACCTGAC
eseO P1	KpnI	GCCGAT**GGTACC**CGGATCCTTTGTCATATCATTGTCTTCCCTCCTG
eseO P2	EcoRI	GTACGCTCGATGCT**GAATTC**AGATGTAGGGGGCGACCATTCC
eseN P1	PstI	GTACGCTCGAG**CTGCAG**CGTCTAGAAAGTTGAGCTGGAAGGTTTTCAGG
eseN P2	HindIII	GCCGC**AAGCTT**CTCTGTCATTAAACGATAAAACGGCTCCTCTCGTAATGCTTG

a Restriction endonuclease sequences included to facilitate cloning are in bold.

### Statistical analyses.

All data analysis was done using the GraphPad Prism 5 software (GraphPad Software, Inc., La Jolla, CA). Data for the norvaline experiment were analyzed by using the unpaired *t* test to compare the treated and nontreated cultures for each effector-CyaA strain. Intracellular replication data were analyzed using one-way analysis of variance with Dunnett’s multiple-comparison posttest. Data for the *in vitro* pH shift experiment were analyzed by using a paired *t* test.
